# Outcome selection of randomized controlled trials in acute stroke: a comparative analysis between traditional Chinese medicine and Western medicine

**DOI:** 10.3389/fmed.2026.1796071

**Published:** 2026-04-10

**Authors:** Xinyi Shi, Sisong Cheng, Guanxiang Yin, Yafei Huang, Congren Zhou, Chengji Liu, Jiayi Zhao, Xiaoling Fu, Xiaohui Zheng, Sisi Chen, Xingchen Liu, Nan Yang, Ying Gao, Xinxing Lai

**Affiliations:** 1 Department of Neurology, Dongzhimen Hospital, Beijing University of Chinese Medicine, Beijing, China; 2 Department of Neurology, Jiujiang Hospital of Traditional Chinese Medicine, Jiujiang, China; 3 Department of Neurology, Zhongshan Hospital of Traditional Chinese Medicine, Zhongshan, China; 4 Institute for Brain Disorders, Beijing University of Chinese Medicine, Beijing, China

**Keywords:** core outcome sets, outcome assessment, randomized controlled trials, stroke, traditional Chinese medicine

## Abstract

**Background:**

Stroke is a serious worldwide health issue. In the management of stroke, traditional Chinese medicine (TCM) is frequently used as an alternative treatment. However, differences in outcome measures between Chinese herbal medicines (CHMs) and Western medicine (WM) trials have hindered progress in evidence-based acute stroke treatment. This study compares outcome measures between CHMs and WM, and aims to provide recommendations for improving TCM-based or integrative pharmaceutical trials in acute stroke.

**Methods:**

This study focused on outcome measures in acute stroke randomized controlled trials (RCTs) published between 2020 and 2024. Seven databases were searched: PubMed, Embase, the Cochrane Library, CNKI, VIP, Wanfang, and Sinomed. We used multiple overlapping strategies to perform a comprehensive search.

**Results:**

Three hundred six RCTs were identified, comprising 171 WM trials and 135 CHMs trials. Ten CHMs trials (7.41%) defined a primary outcome measure, compared to 78 WM trials (45.61%). The most common primary outcome measure was the 90-day modified Rankin scale, used in 44 of 78 WM trials (56.41%) and all 7 of 7 CHMs trials that specified a primary outcome. While 143 WM trials (83.63%) utilized safety outcome measures, only 49.63% of CHMs trials specified safety outcomes.

**Conclusion:**

Potential neglect of primary outcomes and inconsistencies in outcome measures may be expected due to the absence of guidance on core outcome sets (COS) for acute stroke in TCM trials. In order to generate high-quality evidence evaluating TCM as adjuvant therapies in acute stroke, future research should give methodologically rigorous COS development first priority.

## 1 Introduction

Stroke was the second leading cause of mortality, following ischemic heart disease by 2023 (1), and ranked as the fourth cause of disability adjusted life years (DALYs) worldwide in 2021 (2). Across all regions of East Asia, stroke emerged as the primary health burden (3). In China, stroke surpassed other diseases to become the leading cause of years of life lost (YLLs), and the foremost contributor to DALYs in 2017 (4).

Stroke, particularly in its acute phase, results from a complex signaling network (5). Ischemic stroke (IS) accounts for 65.3% of incident strokes, and intracerebral hemorrhage (ICH) for 28.8% (6). Conventional Western medicine (WM) often utilizes reperfusion therapies, such as the administration of intravenous tissue plasminogen activator (tPA) or endovascular thrombectomy (EVT). Although these treatments have demonstrated clinical benefits for acute ischemic stroke (AIS), only fewer than 10% of patients ultimately receive these therapies in real-world clinical practice (7, 8). Furthermore, approximately 40–50% of patients still experience unfavorable functional outcomes or futile recanalization despite the success of EVT (9, 10). In contrast, Traditional Chinese Medicine (TCM) has demonstrated the potential to modulate multiple pathophysiological targets and pathways simultaneously, offering a holistic approach to stroke (11). TCM, with Chinese herbal medicines (CHMs) as its key therapeutic modality, is frequently used as an alternative pharmacological therapy for stroke treatment (12). Data from the first large nationwide registry on stroke general management in China revealed that TCM was widely used among stroke patients, including 71% with IS, and 36% with ICH (13). Growing evidence indicates that TCM has been investigated for stroke through various pharmacological mechanisms (14), and clinical studies have revealed that TCM improves long-term functional outcomes, alleviates neurological deficits, and reduces the recurrence, particularly when combined with conventional treatments in acute stroke, while maintaining a favorable safety profile (15–18).

Randomized controlled trials (RCTs) provide high-quality clinical evidence for evaluating therapeutic interventions in acute stroke, and play a critical role in developing therapeutic guidelines and enhancing care standards (19). However, a critical methodological challenge in this field is the heterogeneity of outcome measures used across clinical trials, particularly between studies rooted in TCM and those based on WM (20, 21). The heterogeneity of the selection of outcomes and the inadequate reporting of primary outcomes across different RCTs can complicate the comparison of trial results and limit the inclusion of many studies in systematic reviews or meta-analyses, which impedes the generation of a higher level of evidence for clinical practice. To address these challenges, the concept of core outcome set (COS) has been proposed. A COS refers to a standardized minimum set of outcomes that should be measured and reported in clinical trials for a specific health condition to reduce heterogeneity in outcome reporting and improve the comparability of evidence across studies (22).

Globally, numerous randomized controlled trials (RCTs) have been conducted in the field of acute stroke. Meanwhile, as the improvement of stroke care has become a national priority, China has already shown great support for conducting and enhancing the quality of stroke-related RCTs. Recently, several high-quality RCTs are emerging in the field of TCM for acute stroke (23–25). In clinical trials, an outcome assesses the effect of an intervention compared to a standard procedure or control (26). Selecting appropriate outcomes for RCTs is essential to ensure execution and interpretation, particularly primary endpoints (27, 28). Rigorous and clearly defined outcome determination is essential to ensure reliable results and their effective implementation into clinical practice, bridging the research-practice gap. Nevertheless, considerable variation in outcome selection and inconsistent reporting of primary outcomes remain common, particularly between CHMs and WM trials. Such limitations diminish the overall value of the research, contribute to resource waste, and hinder progress in developing integrative therapies for acute stroke.

However, the specific differences in outcome measures employed in CHMs and WM trials for acute stroke remain unclear, despite the fact that inconsistent outcome measures for acute stroke have been identified across RCTs. Therefore, through systematically analyzing the outcome measures employed in CHMs and WM trials for acute stroke, this study aims to address this issue. In particular, the study (1) assesses the use and timing of outcome measures in CHMs and WM published pharmaceutical trials, (2) investigates underlying factors contributing to these different outcome measures between CHMs and WM, and (3) provides recommendations on selecting outcome measures for TCM-based or integrative pharmaceutical trials in acute stroke.

## 2 Materials and methods

### 2.1 Search strategy

We comprehensively searched seven databases, including PubMed, Embase, the Cochrane Library, the Chinese National Knowledge Infrastructure (CNKI), the Chinese Science and Technology Journals Database (VIP), the Wanfang Database, and SinoMed. We included published RCTs related to either WM or CHMs for acute stroke from January 2020 to December 2024. We used a variety of keywords in the search strategy, including “cerebrovascular diseases,” “stroke,” and “randomized controlled trials.” The search for Chinese databases was restricted to high-profile journals indexed in the Chinese Science Citation Database (CSCD) and Peking University (PKU) Core Journal Directory. In addition, we used multiple overlapping strategies to perform a comprehensive search. Details of the search strategy are provided in Supplementary material.

### 2.2 Eligibility criteria

Inclusion criteria for eligible studies as follows:

Participants: Adult participants (aged ≥18 years) diagnosed with acute stroke (within 14 days of symptom onset), including ischemic and hemorrhagic strokes. Studies involving only transient ischemic attacks or subarachnoid hemorrhage were excluded.

Interventions: RCTs evaluating CHMs or WM interventions.

Comparisons: Placebo, conventional treatment, or other control.

Outcomes: The selection of outcome measures reported in the RCTs.

Study design: Only RCTs were included. Reviews, systematic reviews, protocols, pilot studies, case reports, observational studies, animal studies, studies without abstracts, conference abstracts, and conference proceedings were excluded. Studies must have been published in English or Chinese (restricted to peer-reviewed journals indexed in the core journals of CSCD or PKU Core Journal) between 2020 and 2024.

Exclusion criteria included studies with unclear randomization methods, unclear onset time, duplicate publications, unavailable full-text reports, or insufficient reporting of outcome measures.

### 2.3 Study selection and data extraction

Pairs of researchers independently selected studies and extracted data according to the eligibility criteria. Any disagreements were resolved through discussion or consulting a third professional researcher. A standardized data collection form was used to systematically extract the data. We extracted: (1) Study characteristics: author, title, publication year, country, journal, sample size, funding source, and registration number; (2) Participant details: stroke type, and duration; (3) Intervention details: type and combination treatment; (4) Outcome measures: primary and secondary outcomes and assessment time. Bibliographic management and duplicate removal were performed using NoteExpress, followed by manual verification.

Some trials reported outcomes such as the total effective rate (TER) and TCM syndrome scores. TER is a composite indicator commonly used to categorize clinical improvement based on predefined criteria, while TCM syndrome scores evaluate symptom severity according to predefined TCM diagnostic frameworks. For outcome measure comparisons, we categorized indicators into the following domains: endpoint event outcome, functional outcome, neurological impairment outcome, imaging outcome, patient-reported outcome, functional impairment outcome, TCM related outcome, safety outcome, and other outcomes.

### 2.4 Assessment of risk of bias

Pairs of researchers independently assessed the risk of bias for included RCTs using the Cochrane risk of bias (RoB), and the results were cross-checked to ensure consistency. The assessment covered the following domains: random sequence generation, allocation concealment, blinding of participants and personnel, blinding of outcome assessment, incomplete outcome data, selective reporting, and other bias. Each item was judged as “low risk,” “high risk,” and “unclear risk” of bias. Any discrepancies between the reviewers were resolved by discussion and in consultation with a third author.

### 2.5 Data synthesis and analysis

Descriptive analyses were used to summarize the frequency and distribution of outcome measures across CHMs and WM. Categorical variables were used to indicate whether a specific outcome measure was reported in each study. Differences in the selection of outcomes between CHMs and WM trials were compared using Fisher’s exact test. A two-sided *p*-value <0.05 was considered statistically significant.

Since a single trial may employ and use multiple outcomes, we also conducted analyses using outcome indicators as the unit of analysis. Outcome measures were categorized into predefined outcome domains, and their frequencies were summarized separately for WM and CHMs trials. Differences in the distribution of outcome domains were assessed using the Pearson chi-square test. Standardized residuals were further calculated to identify outcome domains contributing most to the overall differences between groups, with absolute standardized residuals greater than 2 considered indicative of significant over-representation.

All statistical analyses were conducted using R (version 4.5.1). A Sankey diagram was generated using Origin 2025b (OriginLab) to visualize the comparative distribution of outcome domains and outcome measures.

## 3 Results

**Figure 1:**
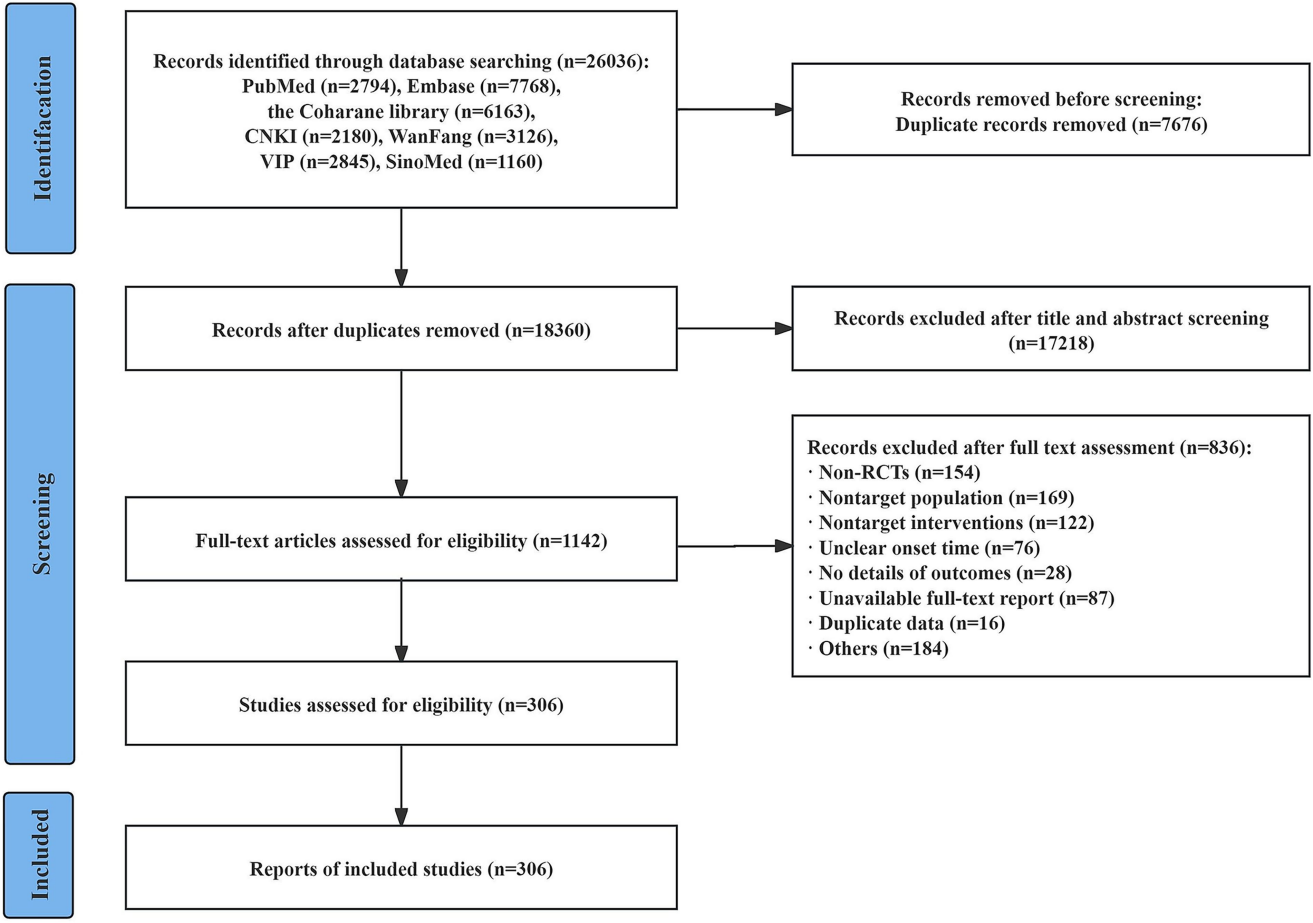
Flow diagram of study selection.

This study synthesized evidence from 306 RCTs, including 171 WM trials and 135 CHMs trials (Figure 1). Of these, 277 IS trials and 26 ICH trials were included, with three trials without classification of stroke subtypes. The median sample size of WM trials was 120 patients (range: 24–11,016 patients; IQR: 84–267 patients), while the median sample size of CHMs trials was 98 patients (range: 40–3,448 patients; IQR: 82–120 patients). Only 5.19% of CHMs trials were registered before research initiation, compared with 44.44% of WM trials. Additionally, in contrast to 51.46% of WM trials, merely 7.41% of CHMs trials were reported in English.

The risk of bias of the included trials was assessed using the RoB. Overall, most studies showed low risk in the domains of random sequence generation, incomplete outcome data and selective reporting, whereas allocation concealment and blinding-related domains were more frequently rated as unclear risk in WM and CHMs trials (Supplementary Figures S1, S2).

### 3.1 Primary outcome

In terms of primary outcome designation, 78 WM trials (45.61%) defined a primary outcome measure, while only 10 CHMs trials (7.41%) set. The difference in the proportion of trials defining a primary outcome between WM and CHMs trials was statistically significant (Fisher’s exact test, *p* < 0.01). In WM clinical trials, 48 trials (28.07%) utilized the modified Rankin scale (mRS) as the primary outcome measure, in which 44 trials designated 90-day mRS; 10 trials (5.85%) selected the National Institutes of Health Stroke Scale (NIHSS); 2 trials (1.17%) employed the Barthel Index (BI), and the remaining studies (*n* = 18) adopted other endpoints. Among CHMs trials, 7 trials (5.19%) used the mRS as the primary outcome measure, all of which were assessed at 90 days, and 3 trials (2.22%) selected the NIHSS. Compared with CHMs trials, WM trials were significantly more likely to use the mRS as the primary outcome measure (Fisher’s exact test, *p* < 0.01). More information is listed in Tables 1, 2.

**Table 1:** Outcome measures in acute stroke RCTs.

Outcome	WM (*n* = 171)	CHMs (*n* = 135)	*p*-value
Primary outcome	78 (45.61%)	10 (7.41%)	<0.01
Modified Rankin scale (mRS)	48 (28.07%)	7 (5.19%)	<0.01
National Institutes of Health Stroke Scale (NIHSS)	10 (5.85%)	3 (2.22%)	0.16
Barthel Index (BI)	2 (1.17%)	0	0.51
Other primary outcomes	31 (18.13%)	1 (0.74%)	<0.01
Secondary outcome	171 (100%)	135 (100%)	1.00
Modified Rankin scale (mRS)	77 (45.03%)	25 (18.52%)	<0.01
National Institutes of Health Stroke Scale (NIHSS)	135 (78.95%)	120 (88.89%)	0.02
Scandinavian Stroke Scale (SSS)	1 (0.58%)	1 (0.74%)	1.00
Canadian Stroke Scale (CSS)	2 (1.17%)	1 (0.74%)	1.00
Barthel Index (BI)	50 (29.24%)	47 (34.81%)	0.32
Activities of Daily Living Scale (ADL)	10 (5.85%)	12 (8.89%)	0.37
Glasgow Coma Scale (GCS)	2 (1.17%)	6 (4.44%)	0.14
Fugl-Meyer Assessment	3 (1.75%)	9 (6.67%)	0.04
Mini-Mental State Examination (MMSE)	11 (6.43%)	7 (5.19%)	0.81
Montreal Cognitive Assessment (MoCA)	7 (4.09%)	8 (5.93%)	0.60
Euro-QOL 5-dimensional Quality-of-life Scale (EQ-5D)	9 (5.26%)	2 (1.48%)	0.12
Short-Form 36 (SF-36)	2 (1.17%)	0	0.51
Stroke Impact Scale (SIS)	3 (1.75%)	0	0.26
Stroke-specific quality of Life (SS-QOL)	2 (1.17%)	2 (1.48%)	1.00
Mortality	13 (7.60%)	1 (0.74%)	<0.01
All-cause mortality	3 (1.75%)	0	0.26
Total effective rate (TER)	8 (4.68%)	2 (1.48%)	0.19
Cerebral infarction Volume	7 (4.09%)	1 (0.74%)	0.08
Hematoma volume	6 (3.51%)	4 (2.96%)	1.00
Composite outcome	3 (1.75%)	1 (0.74%)	0.63
Effective rate	55 (32.16%)	88 (65.19%)	<0.01
TCM Syndrome Score	0	39 (28.89%)	<0.01
Safety outcome	143 (83.63%)	67 (49.63%)	<0.01
Mortality	26 (15.20%)	2 (1.48%)	<0.01
All-cause mortality	17 (9.94%)	1 (0.74%)	<0.01
Symptomatic intracranial hemorrhage (sICH)	32 (18.71%)	5 (3.70%)	<0.01
Bleeding event	32 (18.71%)	1 (0.74%)	<0.01
Serious adverse events (SAEs)	24 (14.04%)	4 (2.96%)	<0.01
Adverse events (AEs)	87 (50.88%)	62 (45.93%)	0.42

RCTs, randomized controlled trials; WM, Western medicine; CHMS, Chinese herbal medicines (CHMs).

**Table 2:** Time frame of the mRS and the NIHSS as primary outcome.

Primary outcome	WM (*n* = 78)	CHMs (*n* = 10)
mRS	WM (*n* = 48)	CHMs (*n* = 7)
7d	1 (2.08%)	0
14d	1 (2.08%)	0
90d	44 (91.67%)	7 (100.00%)
180d	4 (8.33%)	0
NIHSS	WM (*n* = 10)	CHMs (*n* = 3)
24 h	2 (20.00%)	0
72 h	1 (10.00%)	0
7d	4 (40.00%)	1 (33.33%)
14d	3 (30.00%)	3 (100%)
21d	0	1 (33.33%)
30d	1 (10.00%)	2 (66.67%)
90d	1 (10.00%)	0

WM, Western medicine; CHMs, Chinese herbal medicines, mRS, modified Rankin scale, NIHSS, National Institute of Health Stroke Scale.

We also conducted a longitudinal analysis on the designation and selection of the primary outcome measures from 2020 to 2024 (Table 3). Compared to the 2020–2022 period, this study discovered that more studies defined a primary outcome between 2023 and 2024 across both WM and CHMs trials. In 2024, more than half of the WM trials (*n* = 18, 52.94%) utilized the mRS as the primary outcome measure, which was a considerable increase compared to before. Meanwhile, the mRS was used as the primary outcome measure in 20.00% (*n* = 3) of CHMs trials published in 2023 and 18.75% (*n* = 3) of those published in 2024, indicating a significant increase over the 2020–2023 period.

**Table 3:** Comparisons of the choice of primary outcome between 2020 and 2024.

Time period	Trial	Primary outcome	mRS used as primary outcome	NIHSS used as primary outcome
WM	171	78 (45.61%)	48 (28.07%)	10 (5.85%)
2020	37	13 (35.14%)	10 (27.03%)	1 (2.70%)
2021	39	12 (30.77%)	8 (20.51%)	1 (2.56%)
2022	24	6 (25.00%)	4 (16.67%)	2 (8.33%)
2023	37	21 (56.76%)	8 (21.62%)	3 (8.11%)
2024	34	26 (76.47%)	18 (52.94%)	3 (8.82%)
CHMs	135	10 (7.41%)	7 (5.19%)	3 (2.22%)
2020	41	3 (7.32%)	1 (2.44%)	2 (4.88%)
2021	41	0	0	0
2022	22	0	0	0
2023	15	3 (20.00%)	3 (20.00%)	0
2024	16	4 (25.00%)	3 (18.75%)	1 (6.25%)

WM, Western medicine; CHMs, Chinese herbal medicines; mRS, modified Rankin scale; NIHSS, National Institute of Health Stroke Scale.

### 3.2 Secondary outcome

The mRS was used in 77 WM trials (45.03%), and the NIHSS was employed in 135 WM trials (78.95%) for secondary outcome measures. In CHMs trials, 25 trials (18.52%) utilized the mRS, while 120 trials (88.89%) adopted the NIHSS. WM trials used the mRS significantly more frequently than CHMs trials (Fisher’s exact test, *p* < 0.01), whereas CHMs trials were significantly more likely to report NIHSS as an outcome measure (Fisher’s exact test, *p* = 0.02). For the evaluation of activities of daily living, 50 WM trials (29.24%) and 47 CHMs trials (34.81%) showed general agreement regarding the BI, with similar trends observed in the Activities of Daily Living Scale (ADL).

The application of patient-reported outcome measures (PROMs) was relatively infrequent in both WM and CHMs trials. WM trials selected the Euro-QOL 5-dimensional quality-of-life scale (EQ-5D) (*n* = 9, 5.26%), the Short-Form 36 (SF-36) (*n* = 2, 1.17%), the Stroke Impact Scale (SIS) (*n* = 3, 1.75%), and the Stroke-specific quality of life (SS-QOL) (*n* = 2, 1.17%); while CHMs trials utilized the EQ-5D (*n* = 2, 1.48%), the SF-36 (*n* = 0), the SIS (*n* = 0), and the SS-QOL (*n* = 2, 1.48%). Eighty-eight CHMs trials (65.19%) evaluated TER, whereas 55 WM trials (32.16%) evaluated. Additionally, CHMs trials used a unique outcome assessment: 39 trials (28.89%) used the TCM syndrome score. The use of TER and TCM syndrome score differed significantly between WM and CHMs trials (Fisher’s exact test, *p* < 0.01).

### 3.3 Safety outcome

WM trials were significantly more likely to design safety outcomes than CHMs trials (Fisher’s exact test, p < 0.01). One hundred forty-three WM trials (83.63%) set safety outcomes. Of 143 WM trials, 26 trials (15.20%) concentrated on mortality, 17 trials (9.94%) on all-cause mortality, 32 trials (18.71%) on symptomatic intracranial hemorrhage (sICH), and 32 trials (18.71%) on bleeding events. However, fewer than half of CHMs trials (*n* = 67) designated safety outcomes, with the vast majority (*n* = 62, 45.93%) focusing on adverse events (AEs) such as gastrointestinal discomfort and liver and kidney function impairment.

### 3.4 Comparative distribution of outcome domains and outcome indicators

**Figure 2:**
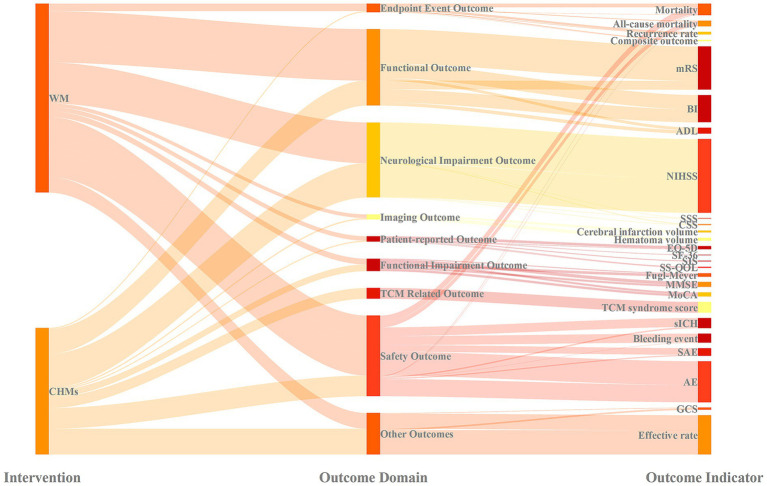
Comparative distribution of outcome domains and outcome measures. WM, Western medicine; CHMs, Chinese herbal medicines; mRS, modified Rankin scale, BI, Barthel Index, ADL, Activities of Daily Living Scale; NIHSS, National Institute of Health Stroke Scale; SSS, Scandinavian Stroke Scale; CSS, Canadian Stroke Scale; EQ-5D, Euro-QOL 5-dimensional quality-of-life scale, SF-36, Short-Form 36; SIS, Stroke Impact Scale; SS-QOL, Stroke-specific quality of life; MMSE, Mini-Mental State Examination, MoCA, Montreal Cognitive Assessment; sICH, symptomatic intracranial hemorrhage; SAE, serious adverse event, AE, adverse event; GCS, Glasgow Coma Scale.

We used a Sankey diagram to clarify the landscape of outcome measures and the different emphasis placed on them in WM and CHMs trials (Figure 2). The distribution of outcome measures was significantly asymmetrical. The main flow of outcome domains was directed toward functional outcomes, neurological impairment outcomes, and safety outcomes. In contrast, endpoint event outcomes, imaging outcomes, and patient-reported outcomes received less attention. This pattern was particularly prominent in CHMs trials, where unique TCM-related outcomes were also utilized. Functional outcomes constituted the most prominent domain, focusing mainly on the mRS and the BI. Neurological impairment outcomes were the second most important category, with the NIHSS being nearly the only outcome measure used here. Safety outcomes had a clear and distinct focus: most data related to AEs and serious adverse events (SAEs), while less attention was given to mortality, all-cause mortality, sICH, and bleeding events.

Using outcome indicators as the unit of analysis, the overall distribution of outcome domains differed significantly between WM and CHMs trials (*χ*^2^ test, *p* < 0.01) (Table 4). Standardized residual analysis showed that endpoint event outcomes, functional outcomes, and safety outcomes were significantly over-represented in WM studies. In contrast, neurological impairment outcomes, TCM-related outcomes, and other outcomes were more frequently reported in CHMs studies.

**Table 4:** Distribution of outcome domains between WM and CHMs studies.

Outcome domain	WM	CHMs	Standardized residual	Over-represented in
Endpoint event outcome	27	4	3.14	WM
Functional outcome	187	91	2.9	WM
Neurological impairment outcome	148	125	–2.17	CHMs
Imaging outcome	13	5	1.08	None
Patient-reported outcome	16	4	1.86	None
Functional impairment outcome	21	24	–1.84	None
TCM-related outcome	0	39	–7.76	CHMs
Safety outcome	218	75	5.89	WM
Other outcomes	57	94	–5.94	CHMs
Overall *χ*^2^ test			*χ*^2^ = 141.98, df = 8, *p* < 0.01	

WM, Western medicine; CHMs, Chinese herbal medicines. Positive standardized residuals indicate higher-than-expected frequencies in WM studies, and negative standardized residuals indicate higher-than-expected frequencies in TCM studies. Absolute standardized residuals greater than 2 are considered significant.

## 4 Discussion

Our study analyzed 306 acute stroke randomized clinical trials published between 2020 and 2024 in English-language databases and selected high-impact medical journals from Chinese databases, including almost all of high-impact international publications. We identified that the selection of outcomes differed significantly between WM and CHMs trials. WM trials more frequently reported endpoint event outcomes, functional outcomes, and safety outcomes, whereas CHMs trials more often reported neurological impairment outcomes, TCM-related outcomes, and other outcomes, which may consequently affect trial quality and reliability.

The primary outcome measure represents the most critical dependent variable analyzed. In trials lacking predefined primary outcomes, there might exist a higher probability that the article emphasizes results supporting study objectives (29). Compared to WM trials (45.61%), only 7.41% of CHMs trials rigorously differentiate primary and secondary outcomes. The majority TCM trials frequently defined multiple endpoints without explicit identification of a primary outcome, introducing challenges in ascertaining core therapeutic efficacy and consequently impeding evidence-based clinical decision-making. Moreover, *a priori* sample size calculations are based on the primary outcome (30). Trials neglecting this may use secondary or alternative outcomes to calculate sample sizes, resulting in statistically underpowered study designs and inability to identify meaningful therapeutic effects. In fact, these methodological deficiencies compromise the internal validity and constrain the external generalizability of TCM trials.

Selecting optimal primary outcomes is a pivotal and challenging phase in clinical trial design. Notably, all CHMs trials that set primary outcomes utilize the mRS or the NIHSS, suggesting the trend to align with international outcome measurements in contemporary acute stroke research, especially in the last 2 years. The mRS is a 7-point ordinal scale describing functional dependence or neurological disability (31), and its 90-day assessment is the dominant primary endpoint for evaluating acute stroke interventions. The NIHSS, frequently employed as an early secondary endpoint in stroke trials, is usually measured at 24 h or 5–7 days post-intervention (32). It quantitatively assesses neurological impairment from 0 to 42, with higher scores signifying more severe neurological impairment (33). Evidence demonstrates 24-h NIHSS could serve as a useful surrogate for 90-day mRS, with potential to decrease sample size demands, trial duration and cost (34).

This study confirms that the mRS remains the predominant primary outcome measure in 2020–2024 acute stroke clinical trials, which is consistent with previous research (35). Additionally, no trend has emerged for the widespread adoption of 24-h NIHSS. Whether 24-h NIHSS can serve as a viable alternative to 90-day mRS in future trials requires further exploration and validation. In addition, the NIHSS timeframe in TCM trials exhibits notable inconsistency, and outcome timeframes heterogeneity is a prevalent issue in TCM trials. Moreover, outcome timeframes especially in TCM trials are often ambiguously defined, frequently omitting clearly definition from baseline to specific follow-up time, therefore compromising longitudinal efficacy assessments.

Safety outcomes are evaluated to comprehensively assess intervention risks and benefits, and to establish holistic evidence for clinical application, especially in stroke research, where drug-induced severe adverse events such as sICH, hemorrhagic events, or mortality require rigorous assessment. Most TCM studies primarily reported nonspecific adverse events, such as drug allergies, gastrointestinal discomfort or liver and kidney function impairment. TCM research must prospectively define and standardize safety outcome protocols, and comprehensively consider disease mechanisms. Additionally, TCM trial design should concentrate on extending follow-up periods to evaluate long-term safety outcomes.

In this study, outcome measures such as TER and TCM syndrome score were more frequently reported in CHMs trials, which reflects the symptom-based evaluation framework commonly used in TCM clinical practice. Compared to internationally standardized neurological scales like the mRS and the NIHSS, these indicators mainly target symptom remission and patient condition from individualized analytical perspectives. The benefit of TCM outcome measures, especially TCM syndrome scores, is their holistic focus on the patient’s overall symptom complex and constitution type, which may better capture subtle changes in clinical status and patient experience beyond neurological impairments alone. Nevertheless, heterogeneity and poor cross-study comparability result from the absence of standardized criteria and consensus definitions for these outcomes. This restricts the broader acceptability and application of TCM in evidence-based stroke management by impeding evidence synthesis and integration into clinical practice guidelines. In addition, these outcomes frequently depend on physician interpretation rather than objective measures, which increases the risk of bias and reduces reproducibility. To address these limitations, standardized quantitative criteria or scales for TCM symptom evaluation need to be established, and objective tools (e.g., precision instruments for digital analysis of tongue/pulse characteristics) should be integrated to fulfill requirements for quantifiable endpoints.

Comparatively, PROMs used in WM trials offer benefits of capturing patient-centered outcomes that align with contemporary emphasis on health-related quality of life. Unlike PROMs, which primarily assess quality of life and patient-perceived functioning with standardized instruments, TCM efficacy rates do not consistently reflect patients’ subjective experience or functional status. The outcome assessment often relies on researchers’ subjective judgments, reducing study objectivity and credibility due to their individualized diagnosis (36). In fact, some outcome indicators have ceiling effects, so apparent “good” outcomes may not match patient or family expectations (37). It is of great necessity that stroke clinical trials should use PROMs, such as EQ-5D, SF-36, SIS, and SS-QOL, which could collect the health status directly from patients without external interpretation, and capture some aspects not easily measured by other common outcome indicators (38, 39). However, PROMs merely played a limited part in the presentation of trial results in a review of acute stroke clinical trials published between 2010 and 2020 (40). Similarly, our study showed a comparable trend over the past 5 years, both in WM and CHMs trials. We recommend greater emphasis on integrating PROMs into trial design and reporting, especially TCM trials focused on individualized patient symptom evaluation.

Selecting an appropriate outcome measure is a key factor that affects the ultimate value of clinical study results for improving the lives of people with health and disability challenges (25). This study identifies common problems in clinical trials, such as non-standardized design and significant heterogeneity in outcome indicator selection, especially in TCM trials. Consequently, despite considerable RCTs on TCM, many of them are unable to generate clinically meaningful evidence sufficient to support the inclusion in clinical guidelines. As a result, it is essential to improve evaluation methodologies to ensure they not only align with the unique features of TCM but also international standards (41). One potential strategy to address this issue is the development of a standardized COS for TCM trials in acute stroke in the future. Importantly, establishing a TCM stroke COS is a crucial step toward advancing the internationalization of TCM. This will be consistent with the global trend toward integrative medicine, where complementary therapies are rigorously evaluated for safety and efficacy. As clinical trials increasingly advocate incorporating PROMs (42), developing the TCM COS for acute stroke should leverage the advantages of TCM to capture patient-perceived wellbeing and symptom changes. The subsequent integration of the TCM-specific patient-reported outcome (PRO) instrument into the COS will enhance the interpretability, inclusiveness, and patient relevance of stroke outcomes in TCM trials (43).

Our study is unique in clarifying specific disparities in outcome measures used in WM and TCM clinical trials for acute stroke, and providing evidence-based recommendations for outcome measure selection in TCM-based or integrative pharmaceutical trials. This study compared the outcome indicators of acute stoke between CHMs trials and WM trials, developing a comparative example of cross-cultural medical methodology. We identified distinctive indicators based on TCM’s unique concepts, and its shortcomings compared with WM. In the context of globalization, traditional medical systems worldwide also face challenges in balancing evidence-based practices with culturally relevant outcome measures. These traditional medical systems should adapt to modernization and standardization while preserving their core essence. This study provides a comparative example of cross-cultural medical methodology, as varied therapy approaches are required to increase the therapeutic possibilities for complex diseases like stroke. We believe that the methodology in this study clarifies unique outcome indicators and enables traditional medicine to align with modern medicine. Meanwhile, a well-recognized set of COS can guide the design and conduct of future clinical trials, laying the foundation for cross-cultural medical integration and collaboration.

The study has a few potential limitations. For the RCTs sourced from the Chinese database, we exclusively utilized the studies included in the CSCD and the PKU Core Journal. Many RCTs published in Chinese journals lacked adequate descriptions of randomization, as some investigations indicated that merely 6.8% of these RCTs were judged to be authentic randomized trials (44). The CSCD and the PKU Core Journal contain the most authoritative studies in China, and we attended to identify and screen high-quality studies recognized within the academic community for analysis. Hence, potential publication bias may exist in the study. However, this study utilized a systematic and inclusive search methodology to ensure the data were identified in authentic RCTs to the greatest extent possible, and to provide evaluation and guidance for the outcomes design and execution in TCM trials.

## 5 Conclusion

Since the absence of recommendations and guidance regarding the COS in TCM trials for acute stroke, the neglect in primary outcome and inconsistencies in clinical efficacy evaluation may be anticipated. Nevertheless, this study also identified that high-quality and rigorous RCTs of TCM have increasingly emerged, aligning with the choice of outcome measures used in WM trials. Future studies should design and implement optimal outcome measures grounded in clinically validated methodologies, thereby providing high-quality evidence to assess the integration of TCM interventions as supplementary therapies in the future acute stroke management. In addition, the development of a COS specifically for TCM trials in acute stroke may improve the comparability and interpretability of future stroke trials.

## Data Availability

The original contributions presented in the study are included in the article/Supplementary material, further inquiries can be directed to the corresponding authors.

## Glossary

ADL: Activities of Daily Living Scale
AE: Adverse event
AIS: Acute ischemic stroke
BI: Barthel Index
CHMs: Chinese herbal medicines
CNKI: Chinese National Knowledge Infrastructure
CSCD: Chinese Science Citation Database
CSS: Canadian Stroke Scale
DALYs: Disability adjusted life years
EQ-5D: Euro-QOL 5-dimensional quality-of-life scale
EVT: Endovascular thrombectomy
ICH: Intracerebral hemorrhage
IS: Ischemic stroke
GCS: Glasgow Coma Scale
MMSE: Mini-Mental State Examination
MoCA: Montreal Cognitive Assessment
mRS: modified Rankin scale
NIHSS: National Institute of Health Stroke Scale
PKU: Peking University
PROMs: Patient-reported outcome measures
RCTs: Randomized controlled trials
SAE: Serious adverse event
SF-36: Short-Form 36
sICH: symptomatic intracranial hemorrhage
SIS: Stroke Impact Scale
SS-QOL: Stroke-specific quality of life
SSS: Scandinavian Stroke Scale
TCM: Traditional Chinese medicine
TER: Total effective rate
tPA: tissue plasminogen activator
VIP: Chinese Science and Technology Journals Database
WM: Western medicine
YLLs: Years of life lost
