# Supplementary material for: Outcome selection of randomized controlled trials in acute stroke: a comparative analysis between traditional Chinese medicine and Western medicine
Source: Front Med (Lausanne). 2026 Apr 10;13:1796071. doi: 10.3389/fmed.2026.1796071 (PMC13105872; doi:10.3389/fmed.2026.1796071)

Supplementary Material

**Title:** Outcome Selection of Randomized Controlled Trials in Acute Stroke: A Comparative Analysis Between Traditional Chinese Medicine and Western Medicine

- **Section I**

Searching strategies

- **Section II**

Supplementary Figure 1. The risk of bias graph for Western medicine trials.

Supplementary Figure 2. The risk of bias graph for Chinese herbal medicines trials.

**Section I. Searching strategies**

**Searching Strategies (PubMed)**

| **Search number** | **Query** |
| --- | --- |
| **1** | (((((((stroke[MeSH Major Topic]) OR (cerebral hemorrhage[MeSH Major Topic])) OR (cerebral infarction[Title/Abstract])) OR (cerebrovascular accident[Title/Abstract])) OR (cerebrovascular apoplexy[Title/Abstract])) OR (brain hemorrhage[Title/Abstract])) OR (parenchymal hemorrhage[Title/Abstract])) OR (intracerebral hemorrhage[Title/Abstract]) |
| **2** | (((((randomized controlled trial[Title/Abstract]) OR (randomized[Title/Abstract])) OR (placebo[Title/Abstract])) OR (RCT[Title/Abstract])) OR (randomised controlled trial[Title/Abstract])) OR (randomised[Title/Abstract]) |
| **3** | (((((((((((((((((((((((((((((((case report[Title]) OR (meta-analysis[Title])) OR (review*[Title])) OR (systematic review[Title])) OR (subanalysis[Title])) OR (rationale[Title])) OR (protocol[Title])) OR (pilot[Title])) OR (guideline*[Title])) OR (case-control*[Title])) OR (cross-sectional[Title])) OR (retrospective[Title])) OR (registry[Title])) OR (letter[Title])) OR (machine learning[Title])) OR (rat[Title])) OR (mouse[Title])) OR (pathway[Title])) OR (nurs*[Title])) OR (child*[Title])) OR (training[Title])) OR (exercise[Title])) OR (virtual reality[Title])) OR (robot*[Title])) OR (diet*[Title])) OR (yoga[Title])) OR (aerobic[Title])) OR (bridging[Title])) OR (thrombectomy[Title])) OR (surgery[Title])) OR (traumatic[Title])) OR (subarachnoid[Title]) |
| **4** | #1 AND #2 NOT #3  Filters: from 2020 - 2024 |

**Searching Strategies (Embase)**

| **Search number** | **Query** |
| --- | --- |
| **1** | 'stroke':ab,ti OR 'cerebral hemorrhage':ab,ti OR 'cerebral infarction':ab,ti OR 'cerebrovascular accident':ab,ti OR 'cerebrovascular apoplexy':ab,ti OR 'brain hemorrhage':ab,ti OR 'parenchymal hemorrhage':ab,ti OR 'intracerebral hemorrhage':ab,ti |
| **2** | 'randomized controlled trial':ab,ti OR 'randomized':ab,ti OR 'placebo':ab,ti OR 'RCT':ab,ti OR 'randomised controlled trial':ab,ti OR 'randomised':ab,ti |
| **3** | 'case report':ti OR 'meta-analysis':ti OR 'review*':ti OR 'systematic review':ti OR 'analysis':ti OR 'subanalysis':ti OR 'rationale':ti OR 'protocol':ti OR 'pilot':ti OR 'guideline*':ti OR 'case-control*':ti OR 'cross-sectional':ti OR 'retrospective':ti OR 'registry':ti OR 'letter':ti OR 'machine learning':ti OR 'rat*':ti OR 'mouse':ti OR 'pathway':ti OR 'nurs*':ti OR 'child*':ti OR 'training':ti OR 'exercise':ti OR 'virtual reality':ti OR 'robot*':ti OR 'diet*':ti OR 'yoga':ti OR 'aerobic':ti OR 'bridging':ti OR 'thrombectomy':ti OR 'surgery':ti OR 'traumatic':ti OR 'subarachnoid':ti |
| **4** | #1 AND #2 NOT #3 AND [2020-2024)py |

**Searching Strategies (the Cochrane library)**

| **Search number** | **Query** |
| --- | --- |
| **1** | (stroke):MESH OR (cerebral hemorrhage):MESH OR (cerebral infarction):ab,ti,kw OR (cerebrovascular accident):ab,ti,kw OR (cerebrovascular apoplexy):ab,ti,kw OR (brain hemorrhage):ab,ti,kw OR (parenchymal hemorrhage):ab,ti,kw OR (intracerebral hemorrhage):ab,ti,kw |
| **2** | (randomized controlled trial):ab,ti,kw OR (randomized):ab,ti,kw OR (placebo):ab,ti,kw OR (RCT):ab,ti,kw OR (randomised controlled trial):ab,ti,kw OR (randomised):ab,ti,kw |
| **3** | (case report):ti OR (meta-analysis):ti OR (review*):ti OR (systematic review):ti OR (analysis):ti OR (subanalysis):ti OR (rationale):ti OR (protocol):ti OR (pilot):ti OR (guideline*):ti OR (case-control*):ti OR (cross-sectional):ti OR (retrospective):ti OR (registry):ti OR (letter):ti OR (machine learning):ti OR (rat*):ti OR (mouse):ti OR (pathway):ti OR (nurs*):ti OR (child*):ti OR (training):ti OR (exercise):ti OR (virtual reality):ti OR (robot*):ti OR (diet*):ti OR (yoga):ti OR (aerobic):ti OR (bridging):ti OR (thrombectomy):ti OR (surgery):ti OR (traumatic):ti OR (subarachnoid):ti |
| **4** | #1 AND #2 NOT #3 with Publication Year from 2020 to 2024 |

**Searching Strategies (CNKI)**

**TKA=('中风'+'卒中'+'脑梗'+'脑梗死'+'脑栓塞'+'脑血栓'+'腔梗'+'腔隙性脑梗'+'脑血管病'+'脑缺血'+'脑出血'+'颅内出血') AND TKA=('随机'+'对照'+'RCT'+'安慰剂') NOT TI=('鼠'+'通路'+'系统'+'Meta'+'荟萃分析'+'汇总分析'+'集成分析'+'二次分析'+'衍生分析'+'现状'+'进展'+'综述'+'述评'+'医案'+'个案'+'文献计量'+'训练'+'磁刺激'+'介入'+'脑机接口'+'机器人'+'护理'+'音乐'+'虚拟现实'+'减压术')**

**筛选：北大期刊，CSCD期刊，2020-2024年**

**Searching Strategies (WanFang)**

(题名或关键词:("中风" or "卒中" or "脑梗" or "脑梗死" or "脑栓塞" or "脑血栓" or "腔梗" or "腔隙性脑梗" or "脑血管病" or "脑缺血" or "脑出血" or "颅内出血") or 摘要:("中风" or "卒中" or "脑梗" or "脑梗死" or "脑栓塞" or "脑血栓" or "腔梗" or "腔隙性脑梗" or "脑血管病" or "脑缺血" or "脑出血" or "颅内出血")) and (题名或关键词:("随机" or "对照" or "RCT" or "安慰剂") or 摘要:("随机" or "对照" or "RCT" or "安慰剂")) not 题名: ("鼠" or "通路" or "系统" or "Meta" or "荟萃分析" or "汇总分析" or "集成分析" or "二次分析" or "衍生分析" or "现状" or "进展" or "综述" or "述评" or "医案" or "个案" or "文献计量" or "训练" or "磁刺激" or "介入" or "脑机接口" or "机器人" or "护理" or "音乐" or "虚拟现实" or "减压术")

**筛选：北大期刊，CSCD期刊，2020-2024年**

**Searching Strategies (VIP)**

(M=中风 OR M=卒中 OR M=脑梗 OR M=脑梗死 OR M=脑栓塞 OR M=脑血栓 OR M=腔梗 OR M=腔隙性脑梗 OR M=脑血管病 OR M=脑缺血 OR M=脑出血 OR M=颅内出血 OR R=中风 OR R=卒中 OR R=脑梗 OR R=脑梗死 OR R=脑栓塞 OR R=脑血栓 OR R=腔梗 OR R=腔隙性脑梗 OR R=脑血管病 OR R=脑缺血 OR R=脑出血 OR R=颅内出血) AND (M=随机 OR M=对照 OR M=RCT OR M=安慰剂 OR R=随机 OR R=对照 OR R=RCT OR R=安慰剂) NOT (T=鼠 OR T=通路 OR T=系统 OR T=Meta OR T=荟萃分析 OR T=汇总分析 OR T=集成分析 OR T=二次分析 OR T=衍生分析 OR T=现状 OR T=进展 OR T=综述 OR T=述评 OR T=医案 OR T=个案 OR T=文献计量 OR T=训练 OR T=磁刺激 OR T=介入 OR T=脑机接口 OR T=机器人 OR T=护理 OR T=音乐 OR T=虚拟现实 OR T=减压术)

筛选：北大期刊，CSCD期刊，2020-2024年

**Searching Strategies (SinoMed)**

("中风"[标题:智能] OR "卒中"[标题:智能] OR "脑梗"[标题:智能] OR "脑梗死"[标题:智能] OR "脑栓塞"[标题:智能] OR "脑血栓"[标题:智能] OR "腔梗"[标题:智能] OR "腔隙性脑梗"[标题:智能] OR "脑血管病"[标题:智能] OR "脑缺血"[标题:智能] OR "脑出血"[标题:智能] OR "颅内出血"[标题:智能] OR "中风"[摘要:智能] OR "卒中"[摘要:智能] OR "脑梗"[摘要:智能] OR "脑梗死"[摘要:智能] OR "脑栓塞"[摘要:智能] OR "脑血栓"[摘要:智能] OR "腔梗"[摘要:智能] OR "腔隙性脑梗"[摘要:智能] OR "脑血管病"[摘要:智能] OR "脑缺血"[摘要:智能] OR "脑出血"[摘要:智能] OR "颅内出血"[摘要:智能]) AND ("随机"[标题:智能] OR "对照"[标题:智能] OR "RCT"[标题:智能] OR "安慰剂"[标题:智能] OR "随机"[摘要:智能] OR "对照"[摘要:智能] OR "RCT"[摘要:智能] OR "安慰剂"[摘要:智能]) NOT ("鼠"[标题:智能] OR "通路"[标题:智能] OR "系统"[标题:智能] OR "Meta"[标题:智能] OR "荟萃分析"[标题:智能] OR "汇总分析"[标题:智能] OR "集成分析"[标题:智能] OR "二次分析"[标题:智能] OR "衍生分析"[标题:智能] OR "现状"[标题:智能] OR "进展"[标题:智能] OR "综述"[标题:智能] OR "述评"[标题:智能] OR "医案"[标题:智能] OR "个案"[标题:智能] OR "文献计量"[标题:智能] OR "训练"[标题:智能] OR "磁刺激"[标题:智能] OR "介入"[标题:智能] OR "脑机接口"[标题:智能] OR "机器人"[标题:智能] OR "护理"[标题:智能] OR "音乐"[标题:智能] OR "虚拟现实"[标题:智能] OR "减压术"[标题:智能])

筛选：北大期刊，CSCD期刊，2020-2024年

- **Section II**

Supplementary Figure 1. The risk of bias graph for Western medicine trials.


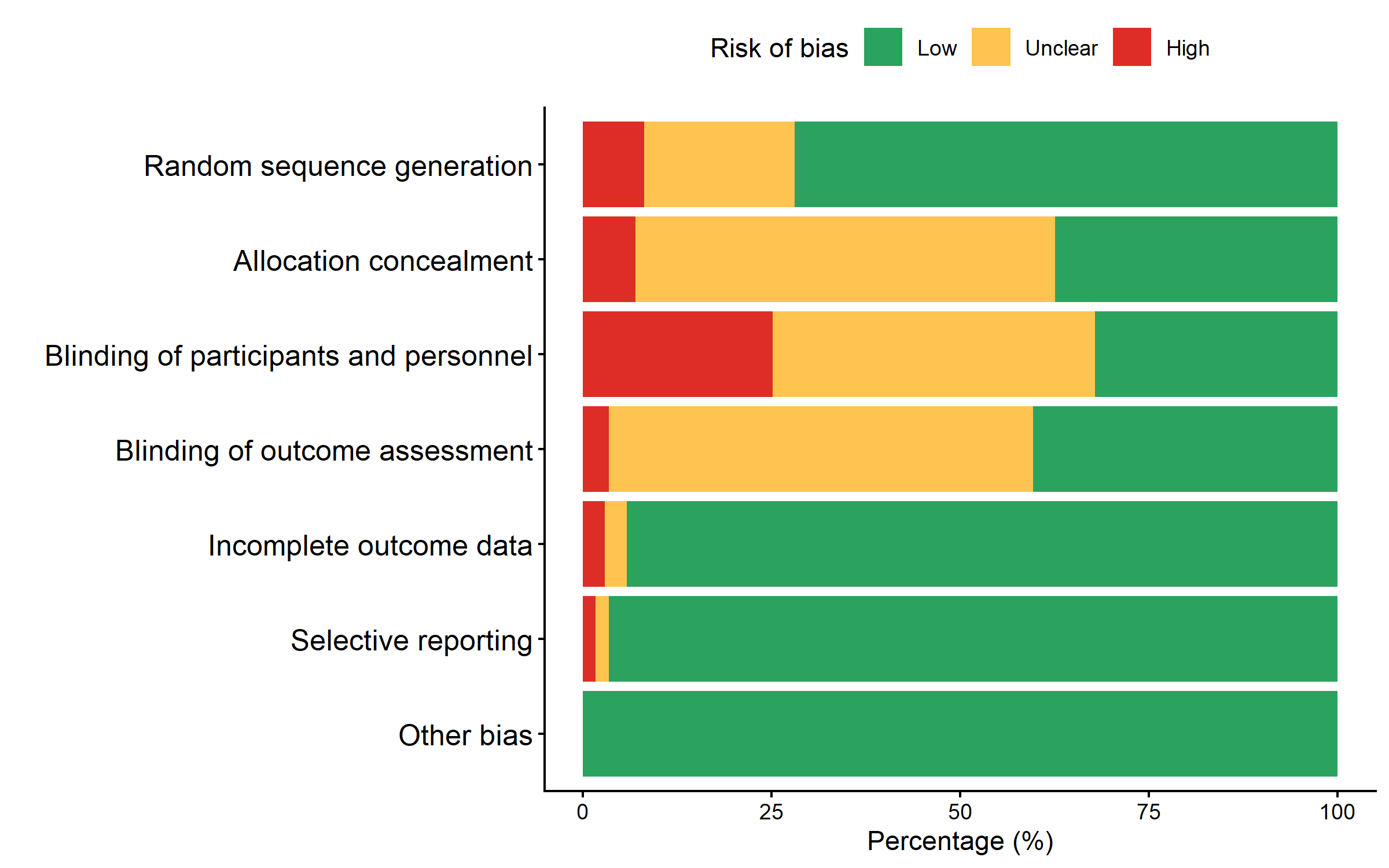


Supplementary Figure 2. The risk of bias graph for Chinese herbal medicines trials.


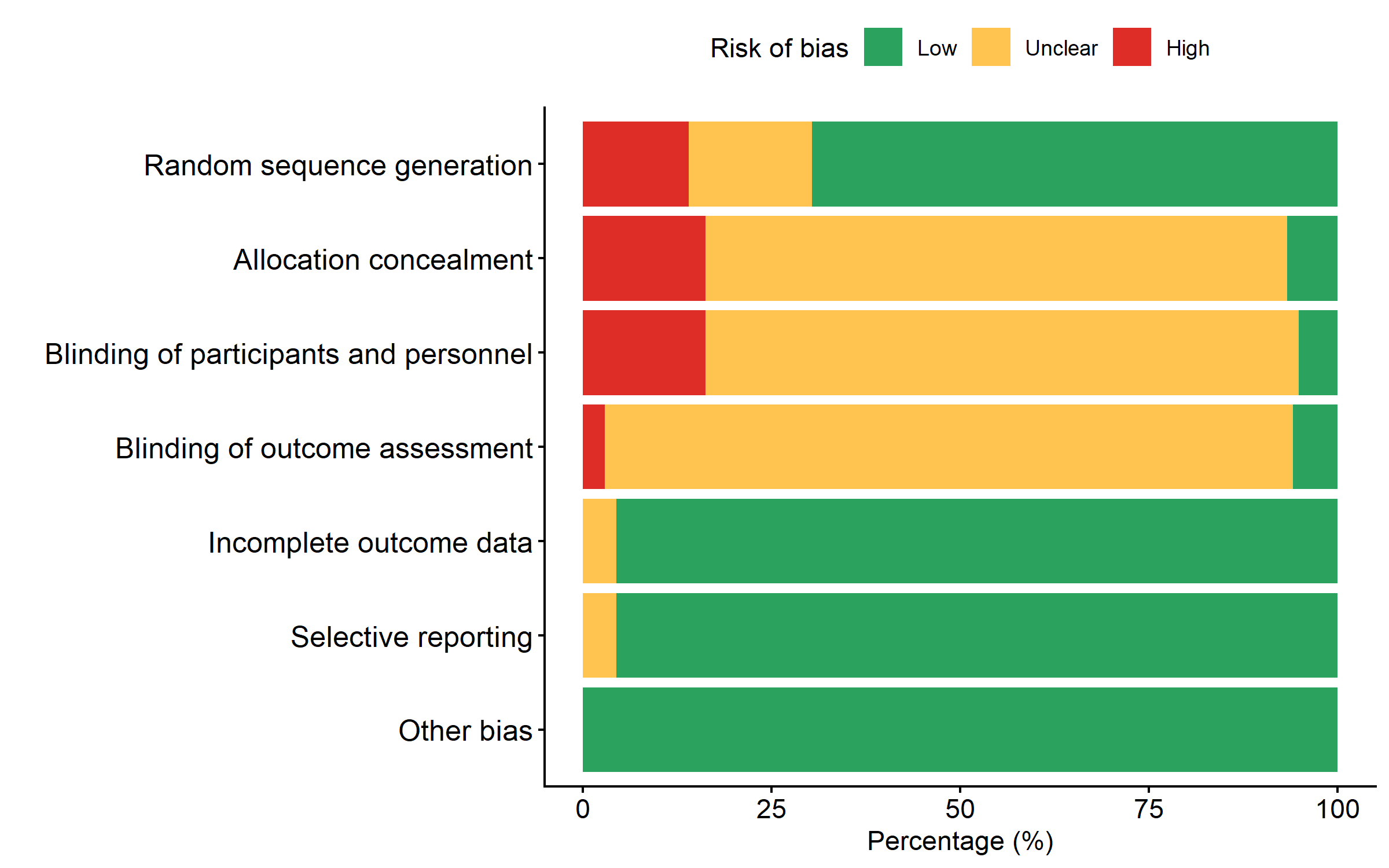

Supplement: Supplementary file 1 [file Supplementary_file_1.docx]
